# Multifunctional performance assessment of waste-based bioplastic wall panels for acoustic thermal and structural efficiency in interior architecture

**DOI:** 10.1038/s41598-025-98326-z

**Published:** 2025-05-05

**Authors:** Hesham H. Awad, Mahmoud Desouki

**Affiliations:** 1 Department of Architectural Engineering, Faculty of Engineering, Menoufeia University, Menoufeia, Egypt; 2 Department of Architectural Engineering, Nile Higher Institute for Engineering and Technology, Mansoura, Egypt

**Keywords:** Bioplastic cladding, Sustainable construction materials, Acoustic insulation, Eco-Friendly architecture, Thermal efficiency, Environmental sciences, Engineering

## Abstract

The study presents a comprehensive evaluation of bioplastic wall cladding units fabricated from organic and plant-based wastes, focusing on their acoustic, mechanical, and thermal performance. The research investigates various configurations, including solid, hollow, and carpet waste-covered tiles, to assess their multifunctional properties. Acoustic testing revealed that carpet waste-covered tiles provide superior sound insulation, achieving performance levels comparable to laminated gypsum boards. Mechanical testing demonstrated sufficient yield strength, flexural strength, and modulus of elasticity, confirming their suitability for non-load-bearing interior applications. Thermal testing highlighted effective insulation capabilities, with linen bark and orange peel configurations showing optimal performance. By integrating recycled materials such as carpet waste, this study addresses critical environmental challenges while enhancing the properties of bioplastic cladding. The findings underscore the potential of these tiles as eco-friendly alternatives to conventional materials, offering architects and designers innovative solutions for sustainable and functional interior design. Future research directions include evaluating long-term durability, fire resistance, and advanced formulations to expand the applications of bioplastic cladding in modern architecture.

## Introduction

The construction industry significantly impacts the environment, contributing to greenhouse gas emissions, resource depletion, and waste accumulation^1^. Plastic-based materials, widely used in construction, especially in architectural finishes and interior cladding exacerbate this issue due to their long degradation time and limited recyclability^2,3^. With increasing awareness of environmental challenges, the demand for sustainable building materials has grown, encouraging the exploration of alternatives that minimize ecological footprints while maintaining functionality. Biomass like wood wastes and industrial byproducts like textiles fibers can be used as fillers or reinforcements in bio composite construction materials^4^.

Bioplastics have emerged as a promising solution in this context. Derived from renewable biological sources such as starches, cellulose, and other plant-based materials, bioplastics offer a sustainable alternative to fossil fuel-based plastics. Bio-based polyurethane systems, demonstrating their applicability as high-performance coatings. Enhancing mortar and concrete formulations, and the use of waste-derived bioplastics for multifunctional cladding panels. Emitting fewer greenhouse gases and leaving no toxic residues upon decomposition make them an appealing choice for environmentally conscious architects^5,6^. The role of bio-composites has been emphasized in enhancing indoor air quality, thermal properties, and acoustic insulation in new buildings and restorations^7^.

Bioplastics have demonstrated versatility in various construction applications, including coatings, claddings, and formworks. For instance, bioplastics could effectively transform the production of lightweight concrete and other construction materials​ by adding them to concrete mixtures and dry premixed mortars^2^. Previous research successfully tested kenaf-reinforced composites, highlighting their potential as wall cladding. with polypropylene to produce bio-composite wall cladding. The results showed that kenaf-reinforced bio composites offered sufficient strength and structural stability for use in non-load-bearing wall applications^8^. Bio-based polyurethane coating as an alternative to petrochemical-based coating emphasized the advantages of using renewable materials like vegetable oils for synthesizing polyols and isocyanates. These bio-based coatings offer impressive physical properties, including flexibility and impact resistance^5^. It is also feasible to use it in facades and interior decorations. Bioplastic combined with starch, which incorporates different starch types blended with agricultural waste and natural fibers, demonstrates promising potential for use as cladding materials for facades and as furnishings. Previous research incorporates potato, corn, wheat, and tapioca starch and tests their performance when blended with various natural additives^9^. A ligno-filled polymer material using polyhydroxyalkanoates (PHA) derived from wood hydrolysates, showcases the potential of these materials in construction, particularly for finishing materials and biodegradable formwork​​. The resulting composite, made from bioplastic and dried solid wood residues, showed promising mechanical strength, suggesting its suitability for construction applications^10^. Recent advances in bioplastics emphasize measurable reductions in emissions and material waste. Compared to conventional plastics, bioplastics reduce lifecycle emissions and support renewable sourcing. Numerous studies highlight the feasibility of using biodegradable polymers in various applications. Table 1 summarizes previous research on bioplastics and bio composites relevant to the present work.

**Table 1:** Reviewed literature.

Title	Main findings	Key Contribution	Relevance to Current Study	Ref.
Production Methods and Applications of Bioactive Polylactic Acid: A Review	Reviews production methods for PLA bioplastics, including surface modification and bioactive compound incorporation.	Explores how bioactive compounds and surface techniques can enhance PLA-based bioplastics.	Surface modification techniques are relevant to enhancing the performance of bioplastic cladding.	^11^
Rethinking Sustainability: A Research on Starch-Based Bioplastic	Evaluates starch-based bioplastics for architectural use; finds tapioca starch most effective.	Identifies effective biopolymers (e.g., tapioca starch) and suggests reinforcement using organic waste.	Shares sustainability goals with the present study and encourages exploration of diverse organic waste sources.	^9^
Use of Filled Bioplastics in Construction	Developed bioplastics using PHAs and wood hydrolysates, with good performance for formworks and surface applications.	Demonstrates the use of wood-derived hydrolysates and bacterial fermentation in construction bioplastics.	Differs in material and application but aligns in promoting eco-friendly construction solutions.	^10^
Bioplastic as a Substitute for Plastic in Construction Industry	Reviews bioplastics’ classifications, properties, and potential in replacing petroleum-based plastics in buildings.	Provides a foundational taxonomy and use-case mapping for bioplastics in architecture.	Broadens the scope of the current research and helps identify bioplastics with better acoustic properties.	^2^
Development of Biocomposite Wall Cladding from Kenaf Fibre by Extrusion Molding Process	Produced cladding panels from kenaf fiber and polypropylene using extrusion; noted benefits and durability challenges.	Introduces extrusion molding for natural fiber composites in cladding applications.	Informed the molding procedures used in the current study.	^8^
A Study of Acoustics Performance on Natural Fiber Composite	Assessed acoustic behavior of natural fibers like kenaf and coconut coir; identified frequency-dependent sound absorption.	Offers data on how various natural fibers influence acoustic properties across frequencies.	Helped in selecting and optimizing materials for acoustic performance in bioplastic panels.	^12^
Panels of Eco-Friendly Materials for Architectural Acoustics	Tested sustainable materials for elasticity and sound insulation, proposing alternatives to synthetic panels.	Benchmarks acoustic performance of eco-materials used in cladding and partitioning.	Informed the acoustic benchmarking and sustainability comparisons in this study.	^13^
Biocomposites for Interior Facades and Partitions to Improve Air Quality	Showed how natural fiber-based bio composites improve thermal/acoustic performance and indoor air quality.	Applies bio composites to enhance indoor health and building comfort parameters.	Provided real-world examples supporting multifunctional cladding in interior applications.	^7^

Despite all the potential advantages of bioplastic, some disadvantages hinder its rapid spread. Bioplastics typically have a higher production cost than traditional plastics, with their price being about twice as much^14^. Bioplastics potentially compete with food resources because they are often made from the same crops, such as corn and sugarcane, used in food production^15^. Additionally, their biodegradability can be a double-edged sword, as it can lead to faster degradation and shorter shelf life in some applications^16^. Various biopolymers exhibit limitations such as significant permeability to water vapor and oxygen, fragility, limited heat resistance, poor mechanical strength, a tendency to degrade easily, and processing challenges^17^.

Noise pollution threatens people’s lives, but architectural treatments can mitigate this by reducing noise emissions^12^. Architectural acoustics studies aim to optimize sound performance in buildings. Integrating issues like speech clarity, excessive noise, and reverberation can be minimized. Modern design trends, materials, and ambient noise levels present challenges to achieving ideal building acoustics^18^. Open spaces and common building materials like concrete can harm acoustic comfort, and fail to meet noise isolation standards^19^. Noise management methods include sound reflection, diffusion, and absorption. Sound reflection involves bouncing sound waves off surfaces, which enhances spaciousness if controlled but causes issues if overused. Sound diffusion scatters waves to prevent direct reflection, while sound absorption converts sound energy into heat, reducing reflection and reverberation. Utilizing sound-absorbing materials can improve sound quality and clarity^20^. Studies show that wall claddings significantly influence interior sound. Adding sound-absorbing claddings improves sound distribution^21^. Studies have focused on developing new cladding materials. Polyester fibers have been extensively studied as sound-absorbing materials for acoustic applications^22^. However, most of the cladding products have been made from fossil fuel-based plastic which encourages us to examine the bioplastic cladding. Attempts have been made to develop wall claddings from bio-based fibers^12^. There are three categories of bioplastics: biodegradable bio-based, biodegradable fossil-based, and non-biodegradable bio-based, the last one is the most environmentally friendly^23^.

Choosing interior cladding as an architectural application would reduce the negative impact of common bioplastic defects for several reasons: Interior cladding is used within buildings, where humidity and temperature are more controlled compared to outdoor settings. This reduces the exposure to conditions that might exacerbate their inherent weaknesses. The vulnerability of bioplastics to degradation from UV light exposure is a lesser issue for interior applications, thereby extending the lifespan and preserving structural integrity. Interior cladding typically experiences less mechanical stress than exterior applications. This helps ensure that the inherent fragility of bioplastics is not a major concern. It also offers more flexibility in terms of aesthetics. Bioplastics can be designed and modified to enhance interior aesthetics. Interior cladding is more accessible for maintenance and replacement if needed. Relying on fiber from non-food sources in bioplastic such as flax bark, fruit peels, nut shells, and egg cartons will reduce pressure on food crops.

Acoustic performance, mechanical resilience, and thermal insulation are critical factors in the selection of interior cladding materials. Noise pollution, a growing concern in urban environments, necessitates effective sound insulation solutions. Simultaneously, the material must possess sufficient structural integrity for practical use and contribute to energy efficiency through thermal insulation.

Compared to previous studies that addressed isolated aspects of bioplastic performance, the current research offers a more integrated assessment. For instance, Gökçe^9^ investigated the mechanical behavior of starch-based bioplastics but did not explore acoustic or thermal properties. Similarly, Abdan et al.^8^developed bio composite cladding from kenaf fibers, focusing on extrusion feasibility and strength, with limited insight into sound or thermal insulation. Roig^7^ and Fontoba-Ferrándiz et al.^13^ emphasized the acoustic and indoor air quality benefits of biocomposites but did not evaluate mechanical robustness. While Safin et al.^10^ successfully incorporated wood waste in bioplastics for surface materials and formwork, their study did not assess indoor applications or acoustic performance. In contrast, this work combines acoustic, thermal, and mechanical testing across varied waste-derived bioplastic formulations, including novel additives like recycled carpet fibers, offering a holistic understanding of material viability in interior wall cladding systems.

This study presents several novel contributions to the field of sustainable construction materials. First, it introduces a holistic evaluation of bioplastic wall cladding by simultaneously assessing acoustic, mechanical, and thermal performance—an approach rarely undertaken in previous research. Second, it explores the use of unconventional organic waste additives such as orange peel, banana peel, nut shells, and recycled carpet fibers, offering an innovative path for material circularity. Third, the study proposes and tests three functional tile configurations—solid, hollow, and carpet waste-covered—demonstrating how design variations impact performance metrics. Finally, the research applies standardized testing protocols (e.g., ISO 10140-4) across dual laboratory settings, providing replicable and statistically supported results. Together, these innovations establish a new benchmark for eco-efficient wall cladding systems suitable for interior architectural use.

## Methods and materials

This study evaluates the acoustic, mechanical, and thermal properties of bioplastic wall cladding units fabricated from organic and plant-based wastes. The methodology consisted of three phases: material preparation, testing, and data analysis. Each phase was designed to ensure a comprehensive assessment of the bioplastic tiles’ performance under standard conditions.

**Fig. 1:**
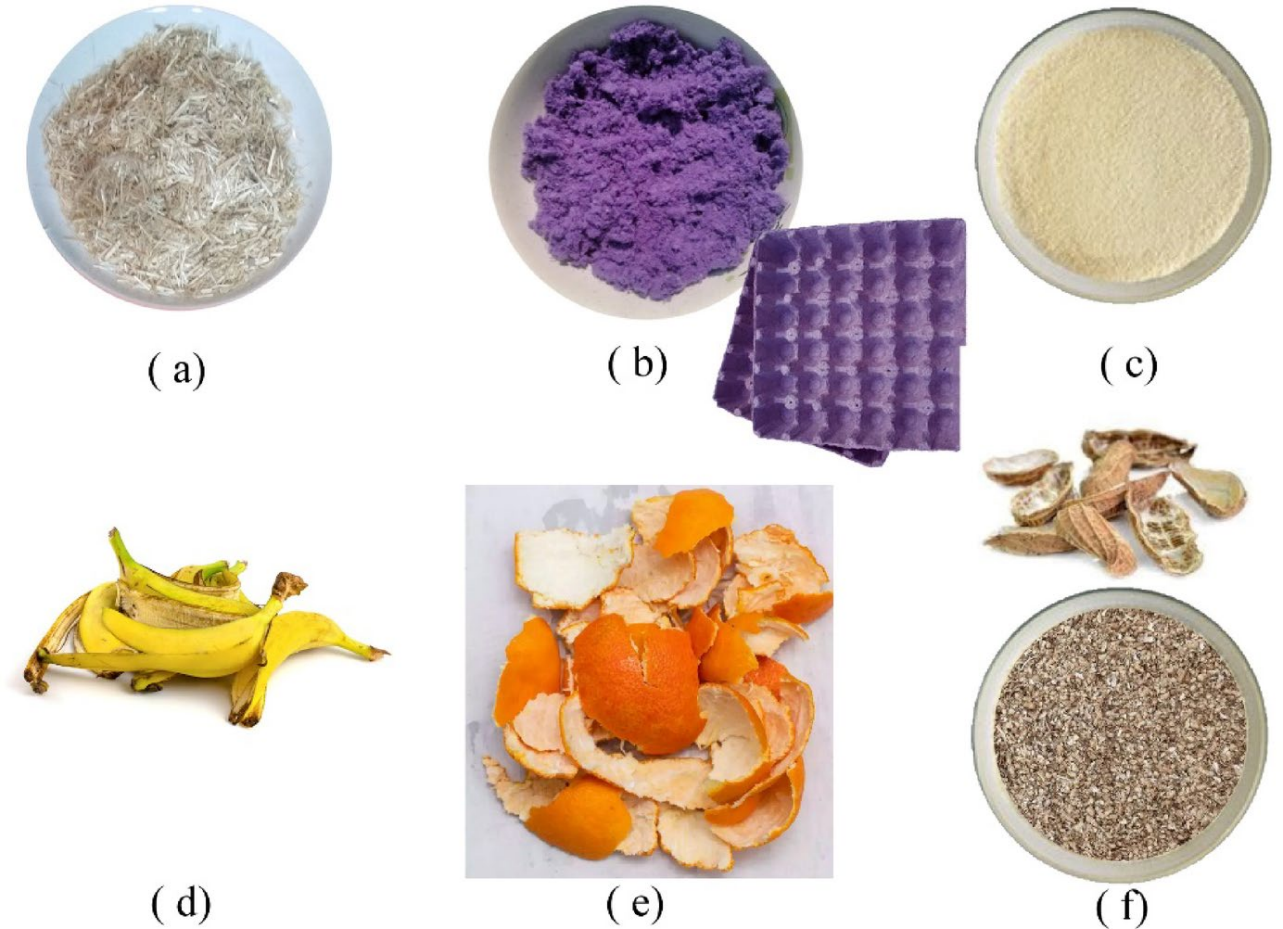
Added waste alternatives.

To produce the bioplastic cladding units, the cornstarch-based method was relied on^24,25^. Cornstarch is characterized by its cheap price compared to other alternatives. All ingredients are combined and stirred in a lab vessel until smooth. The mixture is then heated on medium-low with stirring until it boils and thickens, with food coloring added if desired. After removing from heat, the mixture is spread onto foil or parchment paper inside the mold. Bubbles are removed with a pin. The mixture is left to dry in the air for at least two days, or baked in the oven for 15–30 min. It is checked afterward to ensure it has fully hardened. Table 2 indicates the ingredients. Three sets of bioplastics cladding samples were produced, each consisting of six types of 20 × 20 × 2 Cm tiles: solid tiles, hollow tiles, and tiles covered with carpet waste. All six mixtures shared basic ingredients of distilled water, cornstarch, vinegar, glycerin, and food coloring to differentiate each mixture. However, each set used a different type of waste material to enhance the bioplastic composition as indicated in Table 3. Figure 1 shows the added wastes.

**Fig. 2:**
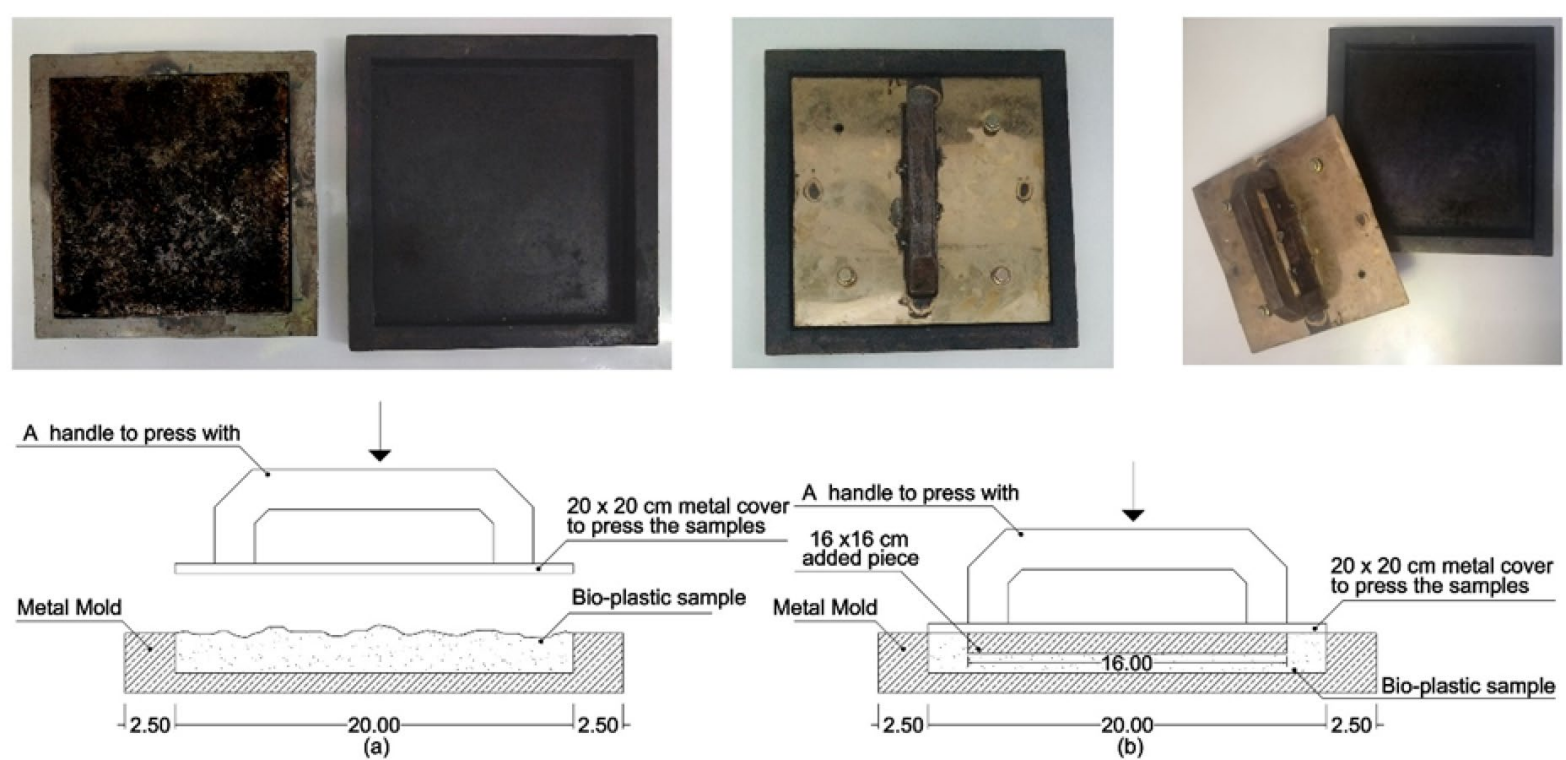
Molds: (**a**) Section showing a casting of solid samples (**b**) Section showing a casting of hollow samples.

**Fig. 3:**
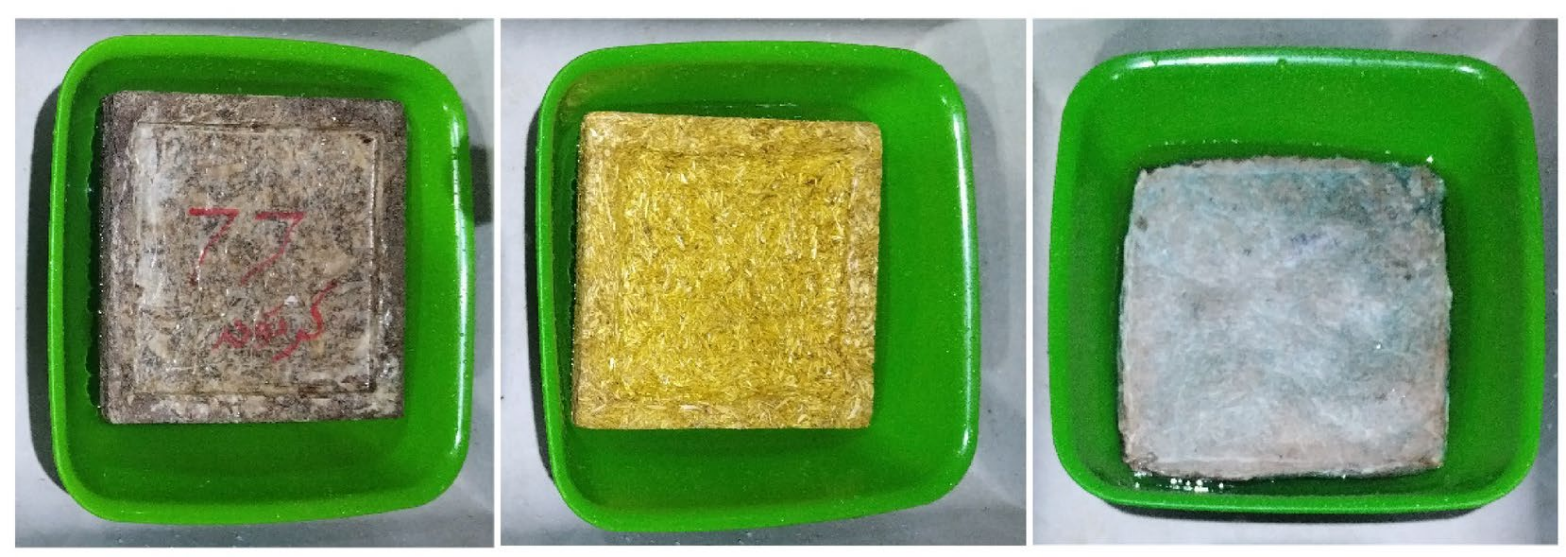
Solid and hollow tiles easily float, indicating low density and mass.

**Fig. 4:**
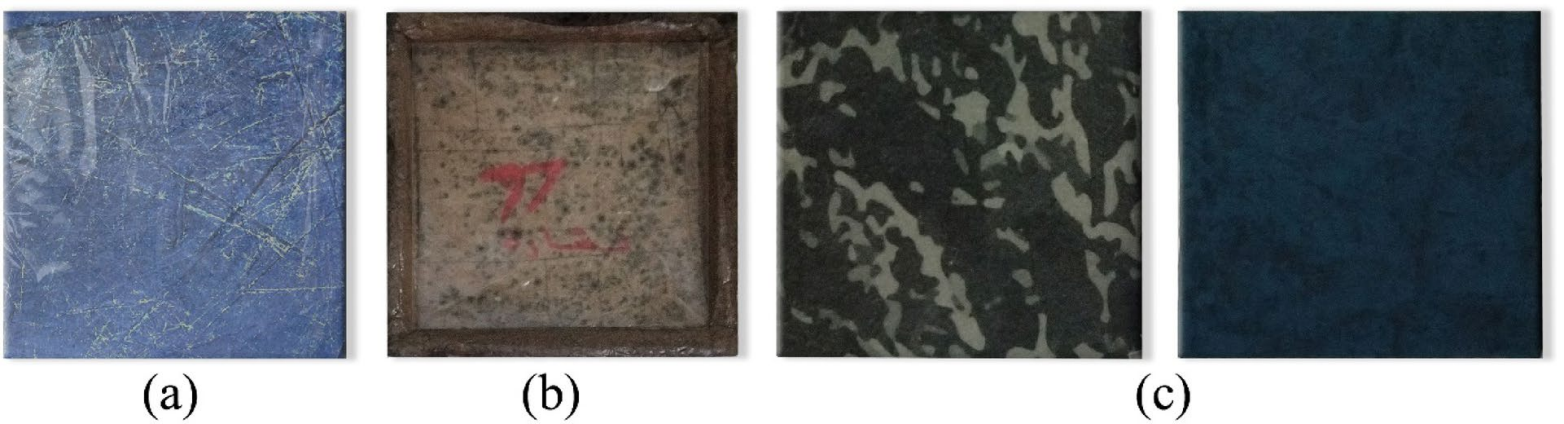
Types of produced tiles: (**a**) Solid (**b**) Hollow (**c**) Covered with carpet waste.

Bioplastic tiles were manufactured following the previously mentioned method, which involved chopping, grinding, and boiling added waste in water before mixing it with basic ingredients. The mixture was poured into six metal molds as shown in Fig. 2, each designed for different tile types with an inner size of 20 × 20 × 2 Cm and an outer size of 25 × 25 × 3 Cm. A metal cover was used to compress the samples, with a 16 × 16 Cm piece added to create hollow tiles. The samples were dried in a regular oven for 15–30 min at 180 °C. A total of 15 units for each set of bioplastic tiles were produced. The samples were placed in sunlight and open air for 7 days to be hardened. Using multiple smaller tiles to cover the test area was necessary due to limitations in mold size, oven capacity, and uniform drying conditions for bioplastic materials. Producing larger tiles would have introduced significant risks of warping, inconsistent thickness, and incomplete curing, which would compromise both the mechanical and acoustic integrity of the samples. Table 4 shows the samples’ basic physical properties. The produced units have low mass and density, so they float on water easily as shown in Fig. 3. Regarding the tiles covered with carpet waste. The inclusion of carpet waste addresses a significant environmental issue, as the global carpet industry produces 12 billion square feet of carpet annually, with only 5% being recycled. The remaining 95% ends up in landfills or is incinerated, contributing to waste management problems^26^. The study explores using carpet waste to cover bioplastic tiles as a potential solution to this issue. Figure 4 shows the three categories of tiles.

**Table 2:** Ingredients to create 100 Gm. Of bioplastics.

Source type	Distilled Water	Substrate	vinegar	Glycerin	Food Coloring
Animal	250 mm (1/4 Cup)	Gelatin 75 gm	4 mm (1 tsp.)	5 mm	2 drops
Algae	250 mm (1/4 Cup)	Agar 25 gm	4 mm (1 tsp.)	2.5 mm	2 drops
Plant	250 mm (1/4 Cup)	Cornstarch 50 gm	4 mm (1 tsp.)	5 mm	2 drops

**Table 3:** Basic ingredients and added waste alternatives.

Basic ingredients									
Distilled Water		cornstarch		vinegar		Glycerin		Food Coloring	
500 mm		100 gm		10 mm		10 mm		2 drops	
**Added waste alternatives**									
**Linen bark**	**Ground egg carton**		**Ground eggshell**		**banana peel**		**Orange peel**		**Ground nut-shell**
30 gm	20 gm		100 gm		90 gm		70 gm		80 gm

**Table 4:** Basic physical properties of tiles.

Sample	Mass (gm)	Volume (Cm^3^)	Apparent density (gm/Cm^3^)
**Linen bark (a)**	180	20*20*2 = 800	0.225
**Ground egg carton (b)**	156	20*20*2 = 800	0.195
**Ground eggshell (c)**	235	20*20*2 = 800	0.294
**banana peel (d)**	210	20*20*2 = 800	0.263
**Orange peel (e)**	188	20*20*2 = 800	0.235
**Ground nut-shell (f)**	203	20*20*2 = 800	0.254

**Table 5:** Sound reduction index (R).

Solid sample	L1 (dB)	L2 (dB)	L1 - L2 (dB)	(*R*) for partition+ samples (dB)
**Linen bark (a)**	120	72	48	51.11
**Ground egg carton (b)**	120	74	46	49.11
**Ground eggshell (c)**	120	75	45	48.11
**banana peel (d)**	120	77	43	46.11
**Orange peel (e)**	120	77	43	46.11
**Ground nut-shell (f)**	120	82	38	41.11
**Hollow sample**	**L1 (dB)**	**L2 (dB)**	**L1 - L2 (dB)**	**(** ***R*** **) for partition+ samples (dB)**
**Linen bark (a)**	120	67	53	56.11
**Ground egg carton (b)**	120	70	50	53.11
**Ground eggshell (c)**	120	71	49	52.11
**banana peel (d)**	120	72	48	51.11
**Orange peel (e)**	120	73	47	50.11
**Ground nut-shell (f)**	120	77	43	46.11
**Carpet waste-covered Samples.**	**L1 (dB)**	**L2 (dB)**	**L1 - L2 (dB)**	**(** ***R*** **) for partition+ samples (dB)**
**Linen bark (a)**	120	32	88	91.11
**Ground egg carton (b)**	120	34	86	89.11
**Ground eggshell (c)**	120	36	84	87.11
**banana peel (d)**	120	37	83	86.11
**Orange peel (e)**	120	37	83	86.11
**Ground nut-shell (f)**	120	39	81	84.11
**Laminated Gypsum board acoustical samples**	**L1 (dB)**	**L2 (dB)**	**L1 - L2 (dB)**	**(** ***R*** **) for partition+ samples (dB)**
**GB. 8.5 mm thick (g)**	120	35	85	88.11
**GB. 9.5 mm thick (h)**	120	34	86	89.11
**GB. 1.2 mm thick (i)**	120	32	88	91.11
**GB. 1.5 mm thick (j)**	120	29	91	94.11

### The acoustical testing

The acoustical testing was conducted in two separate laboratory environments: the first at the Higher Technological Institute in Tenth of Ramadan City, and the second at the Higher Institute for Engineering and Technology in Menoufia. The results obtained from both settings were consistent, showing negligible variation between the two.

The According to ISO 10140-1, the airborne sound insulation test involves using a large speaker to generate approximately 100 dB in a source room divided by a partition into two rooms^27^. The sound level (L1) is measured in the source room and the sound level (L2) in the receiving room. The sound reduction index (R) is calculated using the equation =1−2 + 10 log /^28^, where S is the sample area and A is the sound absorption area. This research used a scaled-down wooden room for testing samples.

To accurately measure the sound reduction index according to ISO 16283-1, specific requirements must be met: using 100 dB or higher of pink noise, ensuring test samples are rectangular or square with a width-to-length ratio between 0.7 and 1, maintaining an air temperature of at least 15 °C in the test room, keeping air humidity between 30% and 90%^29^, and ensuring background noise levels are between 6 and 10 dB.

**Fig. 5:**
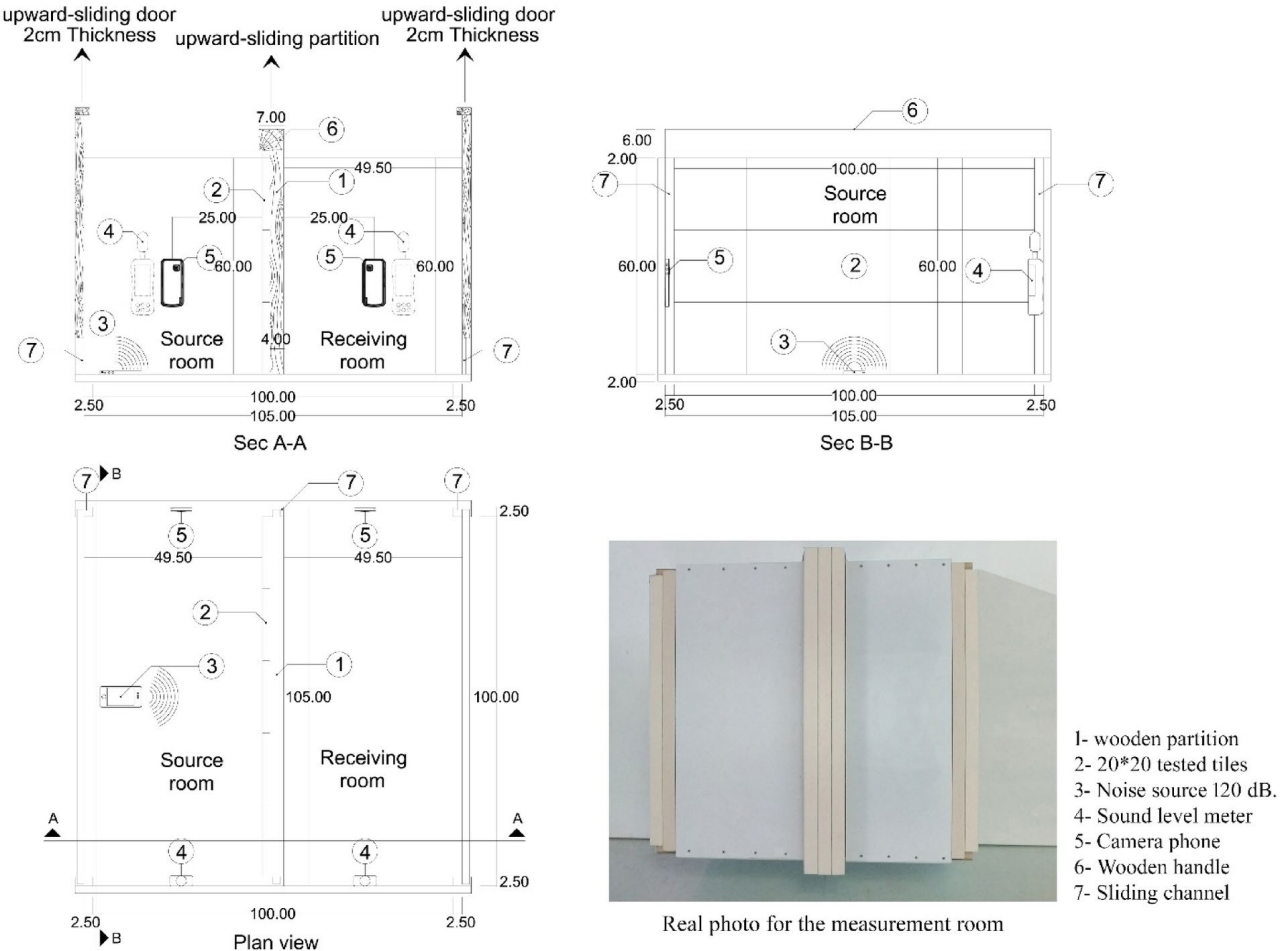
Manufactured measurement room.

The measurement room was manufactured from 2 Cm thick laser-cut plywood, with a 1.05 × 1.05 m base and a height of 0.60 m. It features two upward-sliding doors on opposite faces and a 4 Cm thick wooden partition dividing it into two equal rooms. Rubber pieces were used in joints to minimize sound leakage. The barrier, accommodating 15 tiles of 20 × 20 cm, slides upward for tile stacking. A mobile phone broadcasting pink noise at 120 dB was placed in the source room, and two digital sound level meters and cameras were positioned to record sound readings in both rooms. The Digital Sound Meter TOTAL TETSL01 measures sound from 30 to 130 dB with a dynamic range of 50 dB, sound pressure accuracy of ± 1.5 dB at 94 dB for both 1 kHz and 8 kHz, and a frequency response range of 30 Hz to 8 KHz^30^. Figure 5 indicates the measurement room details.

As for the influence on the sound reduction index (R) for the combined tiles, we acknowledge that interfaces between tiles may introduce minor flanking paths or air gaps, potentially affecting the sound insulation performance. To mitigate this, a tight-fitting arrangement and a wooden clamping frame were used to minimize leakage. The total surface was assembled with care to approximate a monolithic panel as closely as possible.

To conduct the acoustic experiment, pink noise sound waves at a constant intensity of 120 dB were generated across multiple frequencies—125 Hz, 250 Hz, 500 Hz, 1000 Hz, and 2000 Hz—using a frequency generator application on a mobile phone. For each frequency, the sound pressure level in the receiving room was measured using different bioplastic cladding types. Taking the solid bioplastic tile made with linen bark as an example, the measured sound intensity across these frequencies averaged 72.4 dB with a standard deviation of ± 1.02 dB. The low variation between readings reflects the consistency of the test setup and supports the use of 500 Hz as a representative value for calculating the sound reduction index (R) for each material.

In accordance with ISO Standard 10140-4, the sound reduction index should be assessed across a frequency range of 100 to 5000 Hz. Frequencies below 100 Hz are not recommended, as they compromise the accuracy of the sound intensity correlation within the equation.

## Mechanical testing

Mechanical Testing have been conducted in the faculty of engineering material laboratory at Menoufia University.

**Fig. 6:**
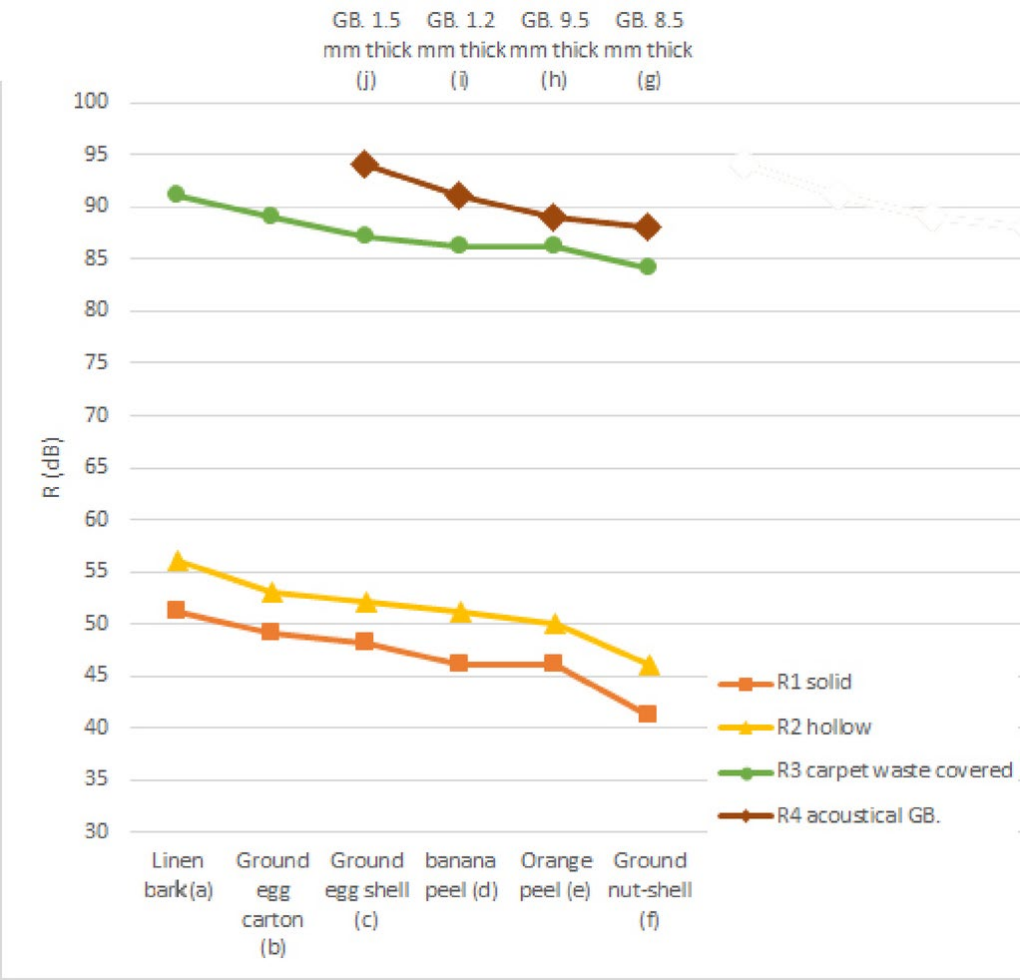
*Sound reduction index for all the samples compared to laminated acoustical gypsum board*.

Mechanical properties, including yield strength, modulus of elasticity, and flexural strength, were measured using standard compression and three-point flexural tests. Cubes of 5 * 5 * 5 Cm and tiles of 20 * 20 * 1 Cm were tested using a compression testing machine and a flexural testing apparatus. A set of 6 types of cubes was cast, using standard metal molds measuring 5*5*5 Cm, shown in Fig. 6a, to test their physical properties.

The cube samples were subjected to a standard Compression testing machine (ELE), shown in Fig. 6b. Figure 6c shows cladding Specimens on the flexural testing machine, where tiles with dimensions of 20*20*1 Cm were tested with a Three-point flexural test at a speed of 2 mm/min, during compression testing, the samples exhibited deformation without structural failure. Interestingly, the deformation resulted in a more aesthetically pleasing appearance, suggesting potential applications in decorative interior elements.

## Thermal testing

**Fig. 7:**
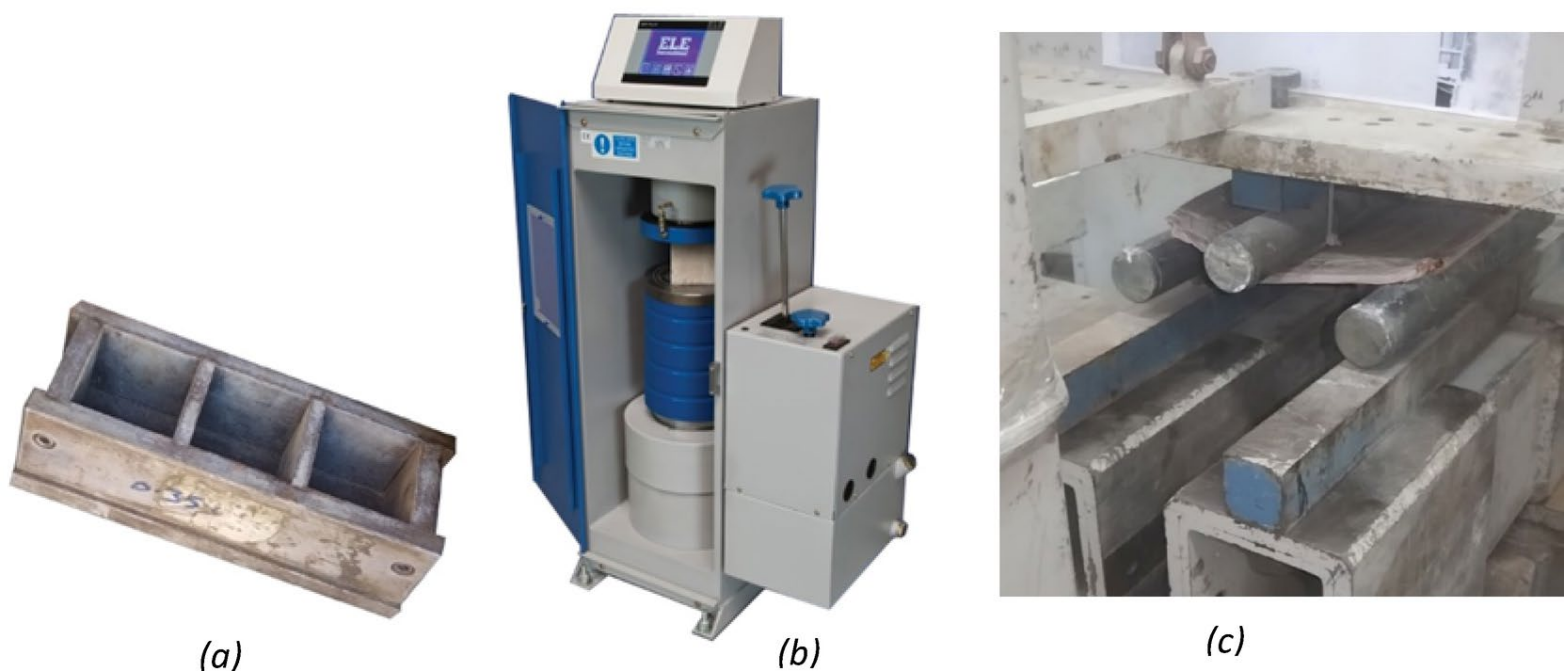
(**a**) Casting metal molds, (**b**) standard Compression testing machine, (**c**) Three-point flexural test for tiles.

Thermal conductivity was assessed using a controlled setup involving an electric heater, brick partitions, and digital thermometers. Tiles were tested to compare their thermal insulation performance to the bare brick partition as the base case. The testing technique was relayed on a previous study^31^ The temperature difference between the inner and outer surfaces of the cladding was recorded every 10 min over an hour. Figure 7 shows the thermal conductivity (K) was calculated using the equation K = (QL)/(AΔT). Where: K is the thermal conductivity in W/m.K, Q is the amount of heat transferred through the material in Joules/second or Watts, L is the distance between the two isothermal planes in meters, A is the area of the surface (m2), ΔT is the difference in temperature in Kelvin or Celsius, Accordingly, we conclude that the thermal conductivity is inversely proportional to the temperature difference (ΔT) between the inner and outer surfaces, when the heat flow rate (Q), the area of the sample (A) and its thickness (L) are constant. thermal insulation, which is the opposite of thermal conductivity, is directly proportional to ΔT.

**Fig. 8:**
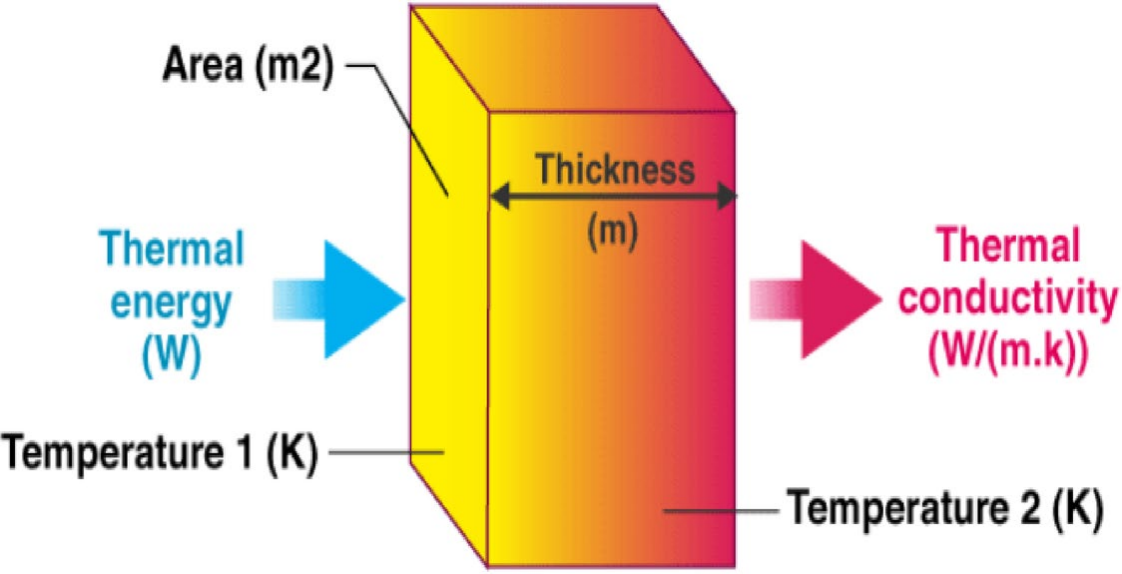
*Thermal conductivity of a material.*

The thermal testing was conducted using a controlled experimental setup to evaluate the insulation properties of bioplastic cladding samples. An electric heater equipped with a thermostat was connected to a heating pad to distribute heat evenly on the tested surface. Temperature measurements were recorded using two PeakTech^®^ P 3695 5-in-1 Digital Mult testers with thermal cables and a FLUKE digital infrared laser thermometer for verification Fig. 8 shows the experiment set.

The experiment involved placing the heating pad between two red brick partitions, ensuring contact with their inner surfaces. The cladding samples were attached to the outer face of one brick partition, while the heater maintained a consistent temperature of 70 °C via the thermostat. Thermal cables were connected to measure temperatures on the outer surfaces of the cladding and the brick partition. Temperature readings were recorded every 10 min over a one-hour duration.

The first stage of the experiment, conducted, involved testing the thermal insulation performance of solid bioplastic cladding samples. The average ambient temperature was 22 °C, with a relative humidity of 48%. The linen bark sample outperformed the other samples. In the second stage, a comparative study to verify the best alternative was conducted under the same conditions between the hollow linen bark tiles (cladding for wall partition 1) and linen bark tiles covered with carpet waste (cladding for wall partition 2). Temperature readings were recorded every 10 min for two hours.

## Results and discussions

Table 5 shows the acoustical experiment results. By substituting into the aforementioned equation (=1−2 + 10 log /), the value of 10 log / can be calculated as follows: = 1.00 * 0.6 = 0.6 m^2^. To calculate , we should know the sound absorption coefficient () for plywood with a thickness of 19 mm at a frequency of 500 Hz, which has a value of 0.15^32^, and accordingly: = (0.6 *1 *2 * 0.15) + (0.495 *0.60 *2 *0.15) + (1.05 *0.495*2 * 0.15) = 2.05 m^2^. So, 10 log /= 10 log (2.05) = 3.11, and it is a constant value in all cases. Sound reduction index (R) for the wooden partition = 120 − 98 + 3.11=25.11.

**Fig. 9:**
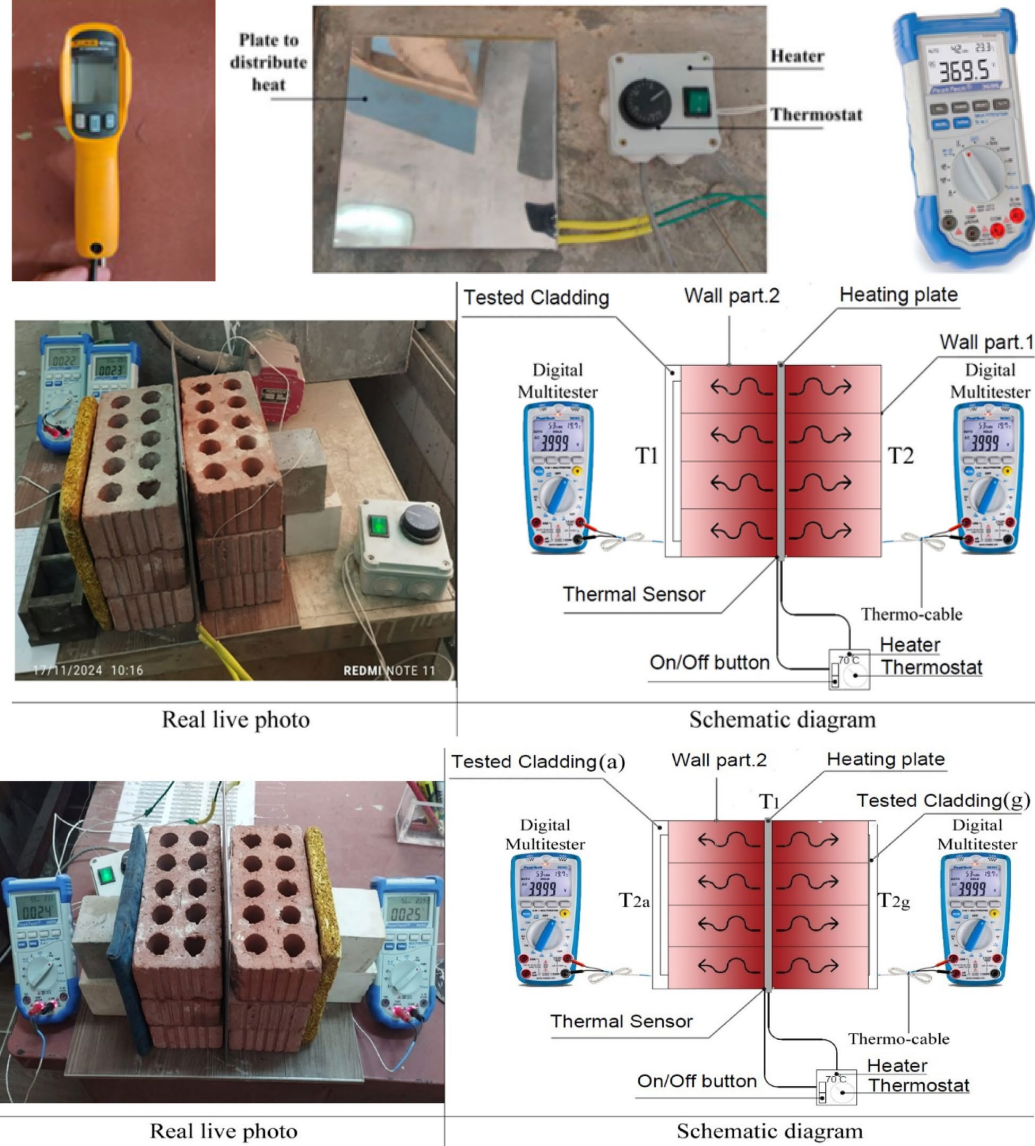
*Real live photo & schematic diagram showing thermal testing.*

The acoustic experiment revealed that carpet waste-covered bioplastic samples significantly outperformed solid and hollow samples in sound insulation, with reductions up to 91.11 dB, comparable to laminated gypsum boards. This significant enhancement can be attributed to the dense, fibrous structure of carpet waste, which introduces both mass and porosity, facilitating the dissipation of sound energy through internal friction and absorption. In contrast, solid bioplastic tiles yielded the lowest performance, with reduction values between 41.11 dB and 51.11 dB. The relatively flat acoustic behavior of these tiles may result from limited internal air cavities, reducing their capacity for energy dissipation. Hollow tiles, however, showed a modest performance increase (up to 56.11 dB), likely due to the presence of enclosed air gaps. This aligns with the acoustic principle that sound travels more slowly and is more easily absorbed in air than in rigid solids. Figure 9 presents the sound reduction index (R) values for all tested configurations. Among the tested compositions, tiles made with linen bark and ground egg cartons stood out due to their lightweight properties and effective acoustic absorption, achieving high R values despite low mass. Their availability and low production cost in Egypt make them viable commercial alternatives for interior acoustic applications, especially in cost-sensitive or sustainable building projects.

When comparing bioplastic tiles in term of physical properties Tiles made with ground egg carton had the lowest weight and density, followed by linen bark, orange peel, ground nut shell, and banana peel, respectively. The highest density was observed in tiles containing ground egg shell. Nevertheless, all samples had densities below that of water, confirming their lightweight classification and suitability for non-load-bearing applications.

Table 6 presents the physical and mechanical properties of the tested bioplastic cubes, including apparent density, yield strength, modulus of elasticity, and flexural strength. The measured densities were consistent with those of the earlier tile configurations, indicating uniformity in material formulation and curing. It increases the reliability of the results. These values confirm that the bioplastic samples possess sufficient structural integrity for use in non-load-bearing interior applications such as wall cladding and decorative panels.

**Table 6:** Yield strength, the modulus of elasticity, and flexural strength of testing cubes.

Sample	Mass (gm)	Apparent density (gm/Cm3)	Yield strength *N*/mm2(MPa)	Yield strength Kg/Cm2 (*N*/mm² value x 10.1972)	The modulus of elasticity (MPa)	The modulus of elasticity Kg/Cm2	Flexural Strength (MPa)
**Linen bark (a)**	29.35	0.234	20	203.8	387.26	3,946.17	29.5
**Ground egg carton (b)**	24.50	0.195	19	193.6	418.84	4,259.42	28.6
**Ground eggshell (c)**	36.24	0.294	24	244.6	292.18	2,977.31	31.7
**banana peel (d)**	32.60	0.260	22	224.2	283.32	2,887.03	30.9
**Orange peel (e)**	29.09	0.232	21	214.0	371.84	3,789.04	29.7
**Ground nut-shell (f)**	31.59	0.253	23	234.4	288.93	2,944.19	30.3

**Fig. 10:**
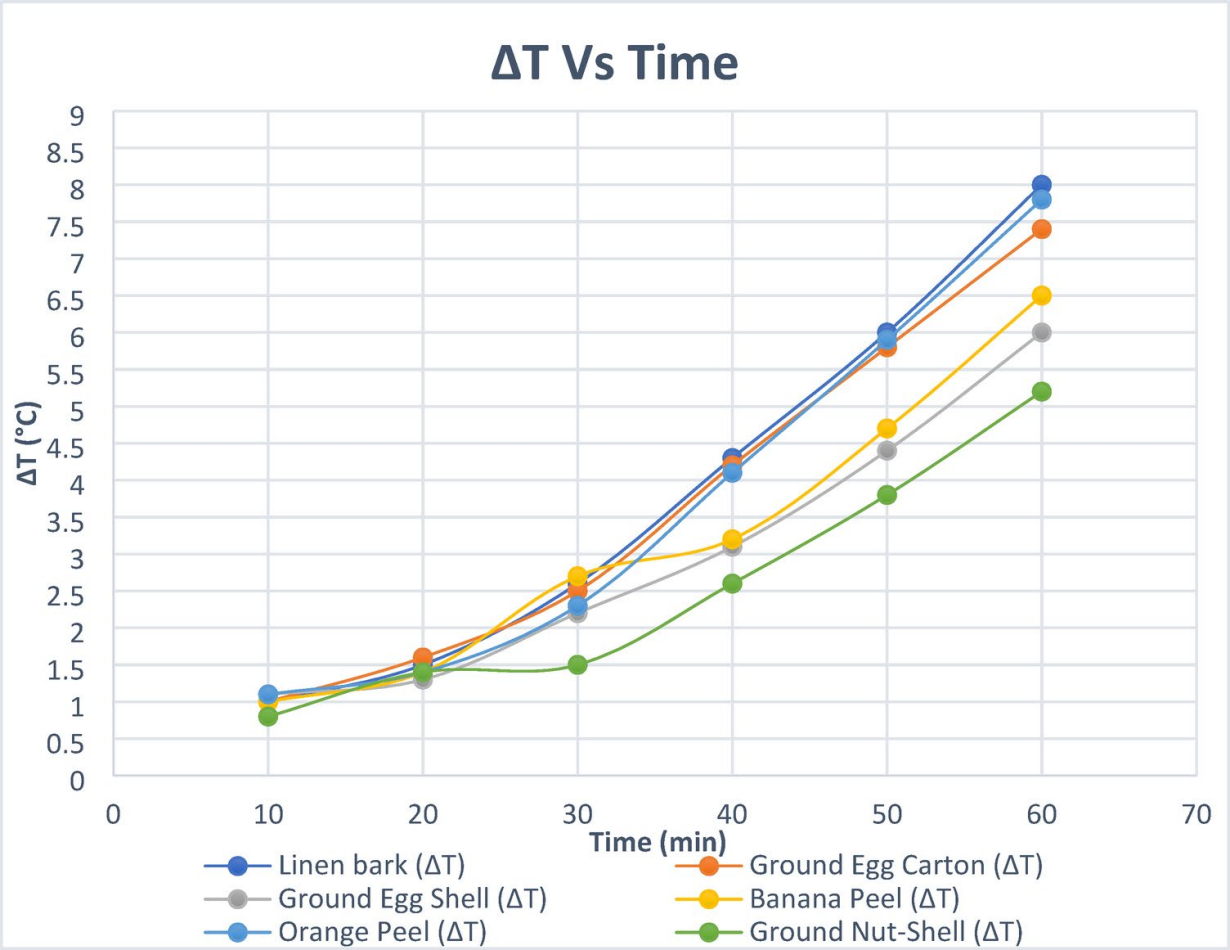
*Shows the thermal insulation abilities of solid bioplastic specimens*.

Egg carton and linen bark achieved the highest modulus of elasticity and compressibility, respectively. Tiles made with orange peel, ground egg shell, ground nut shell, and banana peel followed in descending order. Flexural strength across samples showed minor variation, with ground egg shell tiles achieving the highest and ground egg carton the lowest. Mechanical testing revealed moderate yield strength and flexural performance, suggesting that the materials can withstand minor impact and deformation without cracking or permanent damage Fig. 10 shows the behavior of a sample under the piston of Compression testing machine the shape has distorted but didn’t break. Its potential application in flooring particularly when combined with appropriate surface treatments warrants further investigation. The modulus of elasticity values also indicate that the tiles maintain a balance between rigidity and flexibility an important attribute for panels exposed to handling or minor wall vibrations.

Interestingly, during handling and fabrication, the bioplastic composites appeared to resist ignition and charring, suggesting a potential degree of fire resistance. While this observation is preliminary and not yet supported by formal flammability testing, it opens an important avenue for future research, especially given the growing emphasis on fire-safe materials in sustainable construction.

**Fig. 11:**
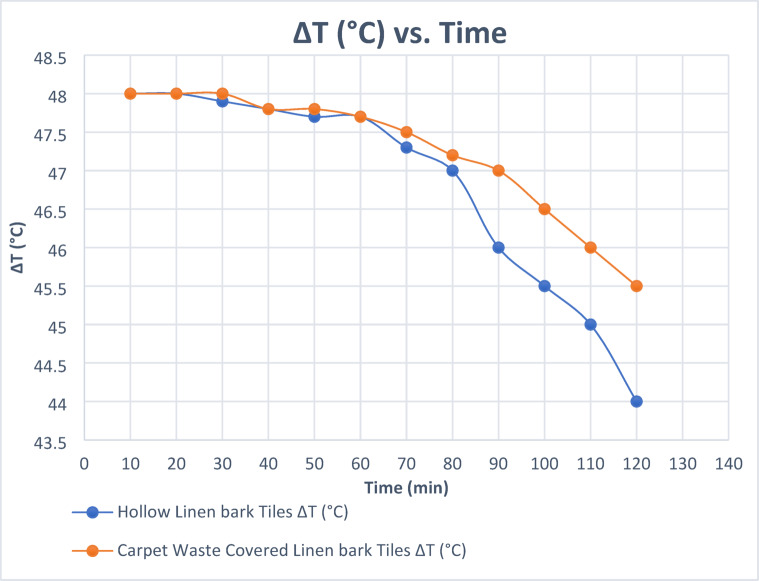
*2nd stage of the experiment*.

Moreover, the controlled deformation patterns observed during compression testing introduced subtle surface modulation and texture, potentially enhancing the aesthetic appeal of the material as shown in Fig. 11. This combination of visual interest and structural resilience positions the bioplastics as compelling options for interior architectural applications, especially in art-driven or sustainability-focused spaces. In addition to wall cladding, the material demonstrates potential for use in interior acoustic panels, ceiling baffles, and decorative furniture skin.

**Fig. 12:**
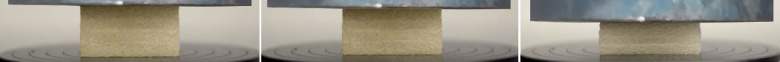
*The compressed sample deformed, distorted, but didn’t break.*

Thermal testing results from Table 7 confirmed that all solid bioplastic samples provided notable thermal insulation, though performance varied depending on the type of added waste. Among them, linen bark and orange peel samples exhibited the highest ΔT values, reaching up to 8 °C and 7.8 °C, respectively, indicating superior insulation efficiency as shown in Fig. 12. This can be attributed to their low thermal conductivity, potential water retention, and porous organic structure, which likely reduced heat transfer across the material. Conversely, the ground nut-shell sample demonstrated the lowest insulation performance, with a ΔT of only 5 °C at 50 min, possibly due to its denser structure and higher thermal conductivity, which enabled more efficient heat transfer.

**Table 7:** Heat transfer time difference between the base case (brick wall) and a covered wall with solid tiles.

Heater temperature 70 °C													
Base case	Linen bark (a)		Ground egg carton (b)		Ground eggshell (c)		banana peel (d)		Orange peel (e)		Ground nut-shell (f)		Time (min)
T2	T1	ΔT	T1	ΔT	T1	ΔT	T1	ΔT	T1	ΔT	T1	ΔT	
23	22	1	22	1	22.1	1.1	22.1	1	22	1.1	22.2	0.8	10
23.5	22	1.5	22	1.6	22.2	1.3	22.2	1.4	22.1	1.4	23	0.8	20
24.6	22	2.6	22	2.5	22.4	2.2	22.8	2.7	22.1	2.3	23.2	1.5	30
26.3	22	4.3	22	4.2	23	3.1	23	3.2	22.2	4.1	23.8	2.6	40
28.1	22.1	6	22.2	5.8	23.7	4.4	23.3	4.7	22.3	5.9	24.3	3.8	50
30.2	22.2	8	22.6	7.4	24	6	23.5	6.5	22.5	7.8	25	5.2	60

**Table 8:** Time lag difference between Hollow bioplastic tiles compared to carpet waste-covered bioplastic tiles.

Heater temperature 70 °C						
Hollow Linen bark tiles (a)			carpet waste-covered Linen bark tiles (g)			Time (min)
T1	T2a	ΔT	T1	T2g	ΔT	
70	22	48	70	22	48	10
70	22	48	70	22	48	20
70	22.1	47.9	70	22	48	30
70	22.2	47.8	70	22.2	47.8	40
70	22.3	47.7	70	22.2	47.8	50
70	22.3	47.7	70	22.3	47.7	60
70	22.7	47.3	70	22.5	47.5	70
70	23	47	70	22.8	47.2	80
70	24	46	70	23	47	90
70	24.5	45.5	70	23.5	46.5	100
70	25	45	70	24	46	110
70	26	44	70	24.5	45.5	120

**Fig. 13:**
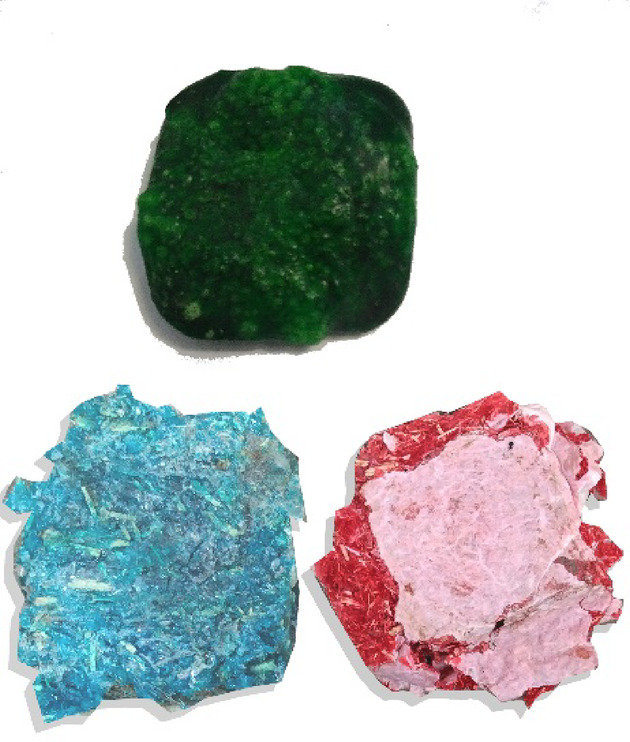
*Compression enhanced* *the aesthetic appeal.*

Further insights from Table 8; Fig. 13 show that carpet waste-covered tiles offered a slightly better thermal barrier than hollow tiles alone. This improvement, while modest (around 0.5 °C on average), suggests that the carpet layer introduced additional resistance to heat flow, likely due to its fibrous, low-conductivity texture. The time lag in temperature rise on the external face also supports this observation, indicating a slower thermal diffusion rate in covered samples.

Collectively, these findings demonstrate that the combination of low-density bio-fillers and surface layering can significantly enhance thermal insulation properties. They also affirm the potential of bioplastic composites to serve not only as cladding but also as thermal regulators in passive building systems, particularly in interior wall applications where energy efficiency is critical.

## Conclusion

This research evaluated the performance of sustainable bioplastic wall cladding tiles produced from various organic and industrial waste additives—namely linen bark, ground egg carton, ground eggshell, banana peel, orange peel, and ground nut shell. Each sample was assessed for its mechanical, thermal, and acoustic behavior, as well as physical characteristics relevant to interior architectural use.

Among all tested compositions, linen bark and ground egg carton tiles emerged as top performers, demonstrating the highest sound reduction indices (up to 91.11 dB) and strong thermal insulation (ΔT of 8.0 °C and 7.8 °C respectively). Mechanically, the same samples showed excellent modulus of elasticity and compressibility, making them suitable for lightweight, non-load-bearing cladding. Although ground egg shell tiles exhibited the highest flexural strength, their density and weight were comparatively higher.

Covering tiles with recycled carpet waste enhanced both acoustic and thermal performance, confirming that surface layering can serve as an effective design strategy. Hollow tile configurations also improved insulation performance due to their air-trapping structure. These findings underscore the versatility of bioplastic cladding for interior applications, offering a combination of environmental benefits, aesthetic appeal, and functional performance. The use of waste materials, including carpet waste, not only addresses significant environmental challenges but also enhances the properties of the bioplastic tiles, making them an eco-friendly choice for modern architecture.

Future developments of this research will focus on several key aspects to enhance both the material performance and its practical applicability. These include evaluating the fire resistance of bioplastic tiles in accordance with relevant building safety codes, conducting long-term durability testing under various environmental conditions (e.g., humidity, UV exposure), and studying the biodegradability and insect resistance of the composites. Using nano-technologies treatments and their effect on the previous issues would be a promising direction.

Additionally, future testing will aim to produce and evaluate single-panel tiles of larger dimensions, as advancements in bioplastic manufacturing techniques allow for more stable and uniform large-scale casting. This will help eliminate the need for assembling smaller tiles, thereby reducing the risk of inter-tile flanking paths or air gaps that can affect acoustic performance. Moreover, advanced sealing strategies will be explored in acoustic testing to further minimize leakage and improve measurement accuracy.

By integrating sustainability with practical functionality, this study positions bioplastic wall cladding as a promising solution to reduce the environmental impact of construction while meeting the demands of modern, eco-conscious design.

## Data Availability

The datasets used and/or analyzed during the current study available from the corresponding author on reasonable request.
